# Using Ecological Momentary Assessment to Examine Predictors of Motivation for Physical Activity in Older Adults

**DOI:** 10.53520/rdhs2025.104152

**Published:** 2025-07-22

**Authors:** Chitra Banarjee, Rui Xie, Chen Chen, Janet Lopez, Ladda Thiamwong

**Affiliations:** 1 College of Medicine, University of Central Florida, Orlando, FL, USA; 2 College of Nursing, University of Central Florida, Orlando, FL, USA; 3 Department of Statistics and Data Science, University of Central Florida, Orlando, FL, USA; 4 Disability, Aging, and Technology Cluster, University of Central Florida, Orlando, FL, USA; 5 Center for Research in Computer Vision, University of Central Florida, Orlando, FL, USA

**Keywords:** digital health, physical function, telehealth, exercise

## Abstract

**Introduction:**

Ecological Momentary Assessment (EMA) involves repeated assessments of patients’ real-time experiences. Smartphone delivery of EMA can monitor healthy behaviors, including physical activity (PA). PA is critical for maintaining mobility and independence in older adults. We aimed to utilize EMA for monitoring motivation for PA, while assessing PA and physical function (PF) as predictors.

**Methods:**

We collected 179 EMA responses from 28 community-dwelling older adults (M_age_ = 72.67±6.55 years, 82.1% female) over one week. Motivation for PA was determined using a single question delivered by a smartphone, PA was monitored using accelerometers, and PF was assessed using dynamic balance (Timed-Up-and-Go) and lower limb power (Sit-to-Stand).

**Results:**

Motivation for PA showed weak correlations with PA (ρ=0.187), dynamic balance (ρ=−0.157) and lower limb power (ρ=0.200). However, mixed effects models revealed that only better dynamic balance (*p*=0.001) predicted increased motivation.

**Conclusions:**

This study revealed the feasibility of monitoring motivation through mobile delivery of EMA, validating the method for future interventions. Evaluation of motivation can assess older adults’ subjective attitudes towards interventions. Additionally, these findings have implications for intervention designs that aim to increase engagement in PA; exercises or methods designed to improve dynamic balance may provide more lasting improvements than focusing on increasing PA alone.

## Introduction

The emerging field of mobile health offers many tools to monitor and assess individual health behaviors. Technology can collect biometric objective information independently of individual input through digital devices such as smartphones, watches, and other sensors. However, understanding subjective information is conventionally limited in clinical research to questionnaires and assessments that are external to the individual’s natural environment. Ecological momentary assessment (EMA) is an emerging tool that provides an alternative method of collecting subjective information related to building healthy lifestyles, such as engaging in physical activity (PA). While recent work has investigated the simultaneous use of EMA and accelerometers for measuring PA in adults aged ≥65 years (older adults),, few have focused on the influence of observed behavior on subjective attitudes.^1–7^ In this paper, we demonstrate the use of EMA to monitor motivation for PA in older adults and its relationship with observed PA and physical function (PF).

Through smartphones and other smart devices, EMA involves sending periodic assessments about behaviors and experiences in real-time, utilized to both collect and deliver information to participants. These dual purposes of EMA are highlighted in the number of different contexts it has been used, including assessment of depressive symptoms, smoking cessation, and PA.^8–10^ Building healthy habits involves intention and action, and EMA offers an opportunity to monitor both. By providing repeated measurements in the natural environment of the participant, it is able to measure real-time intention or motivation for a specific activity, such as PA. Maher and Dunton investigated the link between intention and PA using simultaneous EMA and accelerometry in older adults, and they found that the associations between intentions and action vary over the course of the week.^11^ Other studies indicate that physical inactivity is reliant on both conscious processes, such as goals and attitudes, and non-conscious processes, such as habits that have developed over time.^12^ However, the capacity for PA and PF are likely to influence these processes, but this relationship remains relatively unexplored in older adults.

For older adults, unintentional falls are a leading cause of injury and injury death^13^. Falls have been shown to not only impair activities of daily living but also increase risk of subsequent falls and fear of falling^14–16^. Fall risk prevention is focused around maintaining PA and improving PF.^17–19^ Older adults are recommended to engage in 150 minutes of moderate-to-vigorous PA each week, a target that is widely unmet.^20,21^ This recommendation is in part grounded in the associations between increased PA and improved PF.^22,23^ As such, both variables are critical for screening and interventions for falls in older adults. While both can be measured subjectively using questionnaires, wearable devices and physical performance tests (PPTs) offer more objective measurement of PA and PF, respectively.^24,25^ These objective measures include accelerometers and the Timed-Up-and-Go (TUG), offering similar insights but with greater sensitivity to changes and more functional validity in clinical and research settings.^26,27^

Various intervention models have focused on subjective measures, such as increasing motivation for exercise, by assessing motivational constructs for PA, including self-determination theory, behavior change techniques, and self-monitoring.^28^ Here, we apply the framework of the COM-B (Capability, Opportunity, and Motivation-Behavior) model for behavior modification. In doing so, we explore the interplay between capability (PF), opportunity (directly related to observed PA), and their effects on motivation (as measured through EMA).^29^ Motivation for PA evolves across the lifespan, with intrinsic motivation—fulfilling autonomy, competence, and relatedness—consistently linked to PA.^30^ Specifically in older adults, external motivation, related to receiving an award or avoiding punishment, has been shown to relate to PA by providing health gains^31^. While younger adults often exhibit various motivational drivers, the impact of introjected motivation (e.g., avoiding guilt or shame) in older adults remains inconsistent.^29,32–34^.

This study aims to investigate the role of real-world activity patterns and physical abilities in influencing self-perceived motivation; understanding this relationship can expand and inform future fall interventions available to older adults. We hypothesize that higher PA and better PF will each be associated with greater self-reported motivation, as measured by EMA. Linking objective physical capacity with subjective motivation presents a novel application of EMA in older adults.

## Scientific Methods

### Participants

**Figure 1.**
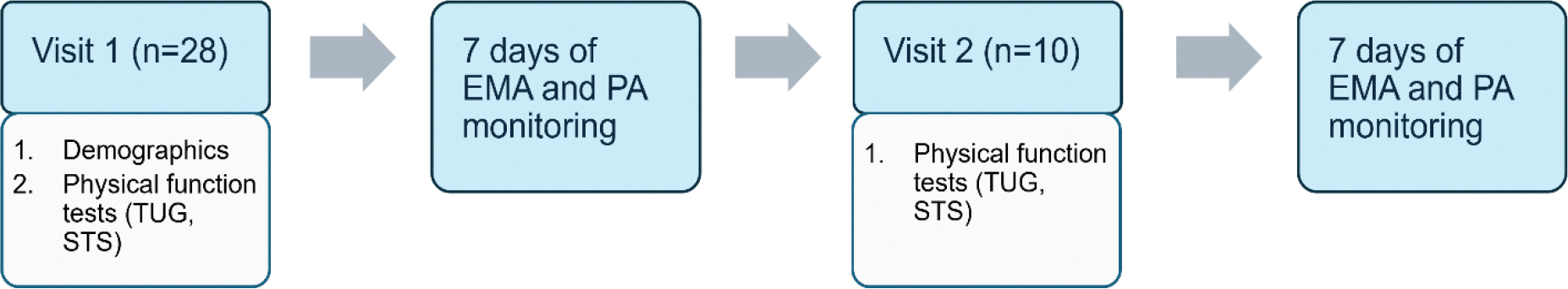
Schematic of Study Protocol. TUG = Timed-Up-and-Go; STS = Sit-to-Stand; EMA = Ecological Momentary Assessments, PA = physical activity.

This longitudinal study was conducted as part of a larger study that is federally funded by the National Institute on Minority Health and Health Disparities (R01MD018025)^35^. The sample consisted of 28 community-dwelling older adults (Mean age = 72.67 ± 6.55 years, 82.1% female, 18 white, 8 African American, and 2 Hispanic older adults). Participants were recruited from community centers who are in low-income households (determined by their eligibility for Section 202 Supportive Housing for the Elderly program) within Orlando, Florida using various strategies, including flyers, word-of-mouth, and collaboration with community partners. The inclusion criteria were that participants must be aged ≥60 years, have no marked cognitive impairment [i.e., Memory Impairment Screen score ≥5),^36^ be able to walk (with or without assistive devices but not requiring assistance from another person), live in their own homes or apartments, and be fluent in English or Spanish. The exclusion criteria were (1) having a medical condition that may preclude engagement in PA (including shortness of breath, dizziness, tightness or pain in the chest, and unusual fatigue at rest or with light exertion) and (2) currently receiving treatment from a rehabilitation facility. Upon enrollment in the study, participants completed a demographics survey. Participants arrived at a local community center to complete physical performance tests and questionnaires. Of the 28 participants, 10 followed up with a second visit and protocol after 2 months (Figure 1). Each participant was guided through the installation process for Catalyst by MetricWire, the platform for EMA collection and given instructions for completing it for the monitoring period of 7 days. They were also fitted with an accelerometer worn on the non-dominant wrist and given instructions on how to wear it during the same monitoring period of 7 days. Given the higher proportion of women in the older adult population frequenting community centers, our study sample exhibited a sex-based bias.

All procedures involving human participants were in accordance with the ethical standards of the institutional and/or national research committee and with the 1964 Helsinki declaration and its later amendments or comparable ethical standards. All study procedures were approved by the University of Central Florida Institutional Review Board (IRB# STUDY00002473). Written informed consent was obtained from all individual participants included in the study.

### Protocol

#### Motivation for PA.

**Figure 2.**
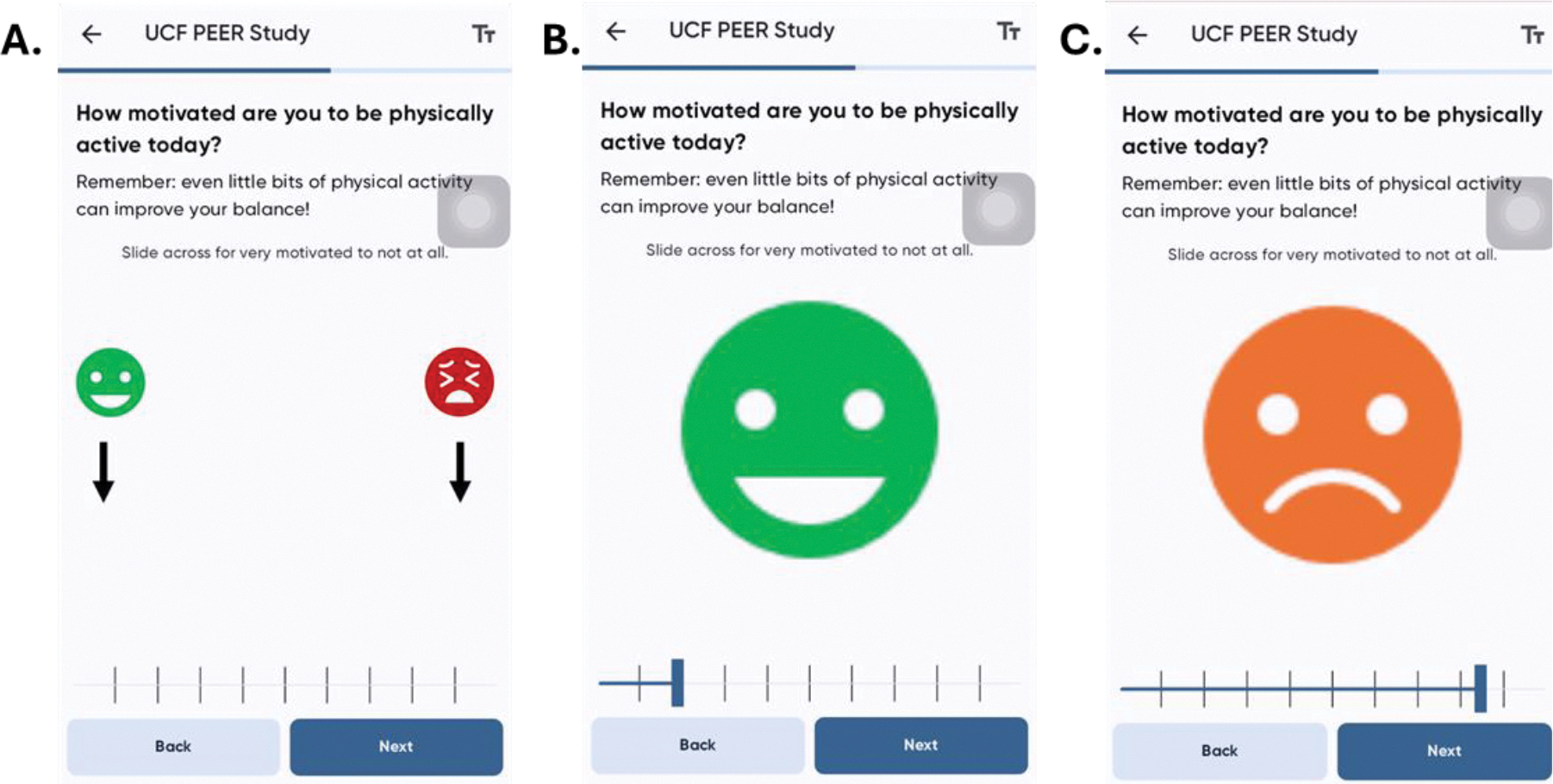
Screenshots of the MetricWire smartphone application with (A) the initial presentation of the question, followed by examples of responses and the images they are associated with (B, C).

Upon beginning the study, participants received an overview of the MetricWire smartphone application and the design of the study. Participants were assisted with the download and setup of the MetricWire app onto their personal smartphones. The app is available for no additional costs to both iOS and Android smartphone platforms. An intake survey asked participants to identify the optimal time in the morning to send a one-question survey to participants about their motivation for PA. Based on that time preference, the MetricWire app delivered a daily EMA survey question using a push notification to participants each day for 7 days (Figure 2). The app also delivered a second push reminder to complete the survey 1 hour after the initial prompt. The question, “How motivated are you to be physically active today?” was designed as an image-based sliding scale, such that high motivation would present with a face with positive affect (Figure 2B) and low motivation would show negative affect (Figure 2C). The item was score 0–100, where lower scores are associated with low motivation. Participants were also presented with a reminder about the benefits of PA. After the 7-day period, the surveys were discontinued. Data was sent directly to MetricWire servers with anonymized participant identifiers when connected to the internet.

#### Accelerometer-Based Physical Activity.

ActiGraph GT9X Link and LEAP (ActiGraph LLC, Pensacola, FL, USA) were used to measure PA levels in participants at each timepoint. The wrist-worn devices are lightweight and small and contain a triaxial accelerometer. The devices were initialized to record data at a sampling rate of 30 Hz (GT9X) and 32 Hz (LEAP) at 1-minute intervals with a dynamic range of ±8 gravitational units (g), as per prior studies.^37^ The ActiGraph LEAP device is a newer model of the GT9X, which expands upon the framework of the GT9X to minimize participant burden and maximize adherence. The ActiGraph LEAP, like the GT9X, has been approved by the Food and Drug Administration for measurement of activity.^38^ Individual participants wore the same ActiGraph model throughout the two timepoints. Participants were instructed to wear it on their nondominant wrist and only remove it near water or undergoing medical imaging for 7 consecutive days, after which the devices were collected from the participants. The ActiGraph GT9X devices have demonstrated high accuracy at distinguishing types of PA based on established cutpoints.^39^

#### Dynamic balance.

Dynamic balance was assessed using the TUG test, performed once at each timepoint.^40^ Before participants completed the TUG test, experimenters instructed and demonstrated the assessment. Participants were instructed to stand up from a chair, walk 3 meters, turn around, walk back to the chair, and sit down again. The measure of dynamic balance was recorded as the time in seconds that the participants take to complete the assessment.

#### Lower limb power.

Lower limb power was assessed using the Sit-to-Stand (STS) test, performed once at each timepoint.^41^ Before participants completed the STS test, experimenters instructed and demonstrated the assessment. Participants were instructed to sit in a chair, place their hands on the opposite shoulder crossed at the wrist, then keep their feet flat and back straight. They are then instructed to rise to a full standing position and sit back down for 30 seconds. The measure of lower limb power was recorded as the number of times that the participant sits in 30 seconds. Participants who unable to stand without using their hands to push off received a score of 0.

### Data processing

#### EMA.

Responses were downloaded using the MetricWire platform as “.csv” files, with their anonymized identifier, date and time of submission, and the response to the prompt. In the case where some participants responded to the question more than one time each day, responses were averaged for each day. The nature of the question produced a skewed response, and the responses were transformed using a logarithmic transformation to normalize the data.

#### PA.

For data analysis, only participants who had at least 10 hours were included. Raw acceleration data was downloaded using ActiLife (GT9X) and CentrePointe (LEAP) and analyzed using R statistical software (R Core Team, Vienna, Austria) to process the “.csv” files. Raw Accelerometer Data Analysis (GGIR), an open-source R-package, was used to process the data to include autocalibration of acceleration signals, non-wear time detection, and calculation of the Euclidean norm of acceleration minus 1 g (ENMO), as previously described.^42,43^ Out of total wear time, the percentage of time spent in sedentary time (ST), light physical activity (LPA), and thi was calculated based on the following cut-off points: (i) ST < 30 milligravitational units (mg); (ii) 30 mg ≤ LPA < 100 mg; (iii) MVPA ≥ 100 mg.^44,45^ This percentage was then averaged across the seven-day period that the accelerometer was worn at each timepoint. After processing, our final dataset consisted of 179 EMA responses with simultaneous PA monitoring. Participants averaged 4.97 responses per week and 71.0% compliance with the EMA notifications.

### Statistical Analysis

All data were stored in a REDCap database managed by the University of Central Florida.^46,47^ All statistical analyses were performed in R statistical software (version 4.3.0, R Core Team, Vienna, Austria). Pearson’s correlation coefficient is appropriate for linear relationships, assumes normality, and achieves efficiency when linearity and normality assumptions are met, whereas Spearman’s rank correlation coefficient is suitable for non-linear relationships, ordinal or non-normally distributed data, and is robust to outliers.^48^ As such, the correlation analysis was performed using Spearman’s correlation coefficient to maintain validity for both normally distributed and non-normally distributed variables. Mixed-effects (multilevel) modeling was utilized with statistical significance level set at 0.05. Linear mixed-effects regression models were fitted using the lmer function in the “lme4” package.^49^ Observations (Level-1) were nested in participants (Level-2) and participants were nested in day of data collection (Level-3). All models contained a random participant intercept and a random day intercept to account for variance associated with those groupings. Three models were presented in the results, each utilizing motivation for PA as the response variable with age and sex as predictors. The models differed in the physical measure used as a predictor, including %MVPA, TUG score, and STS score. For all analyses, a *p* ≤ 0.05 was considered statistically significant a priori. For each model, intraclass correlation coefficients (ICC), marginal R^2^, and conditional R^2^ values are reported. ICCs quantify the proportion of total variance in the outcome explained by clustering (i.e., Participant and Day). Marginal R^2^ represents the variance explained by the fixed effects alone (i.e., the predictors), while Conditional R^2^ includes both fixed and random effects (i.e., overall model fit). Post-hoc power analysis was performed using simulation-based methods to evaluate the fixed effects of interest in each model, based on the observed sample. The analysis was conducted using the simr package in R, with 100 simulations per model.^50^

## Results

Of the EMA responses, participants varied in the number of responses to the daily PA motivation question; 1–3 responses (n = 28), 4–6 responses (n=21), 6–12 responses (n=12). Multiple responses per day were averaged for the analysis. Participants contributed an average of 7.18 (SD = 2.00) responses for the total monitoring period, of which one participant was excluded due only submitting 1 response throughout the week. While there is no accepted standard for an appropriate number of responses, the minimum number of responses from included participants was responses from four days of the week. From the PA data, 43 days from 12 participants were excluded due to insufficient wear time (<10 hours). Table 2 shows the descriptive statistics for the predictor and response variables.

Spearman correlations (Table 2) reveal that motivation for PA as measured by EMA is correlated with observed PA engagement and STS and TUG. Individuals with greater %MVPA and improved dynamic balance (TUG score), and increased lower limb power (STS score) present with greater motivation for PA, suggesting a relationship between action, function, and motivation.

In Table 3, we present the results from the linear mixed-effects models. Motivation for PA was not significantly predicted by %MVPA (p=0.351, Table 3a). Motivation for PA was significantly predicted by TUG score (p=0.001, Table 3b). Motivation for PA was not significantly predicted by STS score (p=0.179, Table 3c). There were no effects of age and sex in all three models.

For Model A, the observed power for %MVPA was 28.00% (95% CI: 10.48%–37.84%). For Model B, the observed power for the TUG score was 93.00% (95% CI: 86.11%–97.14%). For Model C, the observed power for the STS score was 32.00% (23.02, 42.08).

## Discussion

Corresponding with the increasing use of smartphones, EMA is a rapidly developing tool to monitor health behaviors, including PA and SB^3^. The present study implements the use of EMA to understand how real-world activity patterns and physical abilities influence self-perceived motivation. We use correlation analyses and mixed-effects models to evaluate this relationship as measured by a single question assessment of motivation, alongside accelerometer-based measures of PA and PF assessments. In doing so, we find that the PF measure of dynamic balance but not PA or lower limb power affects subjective motivation for PA.

We find weak evidence of observed PA affecting motivation for PA. The results of the correlation analyses found significant correlations between objective measurements of PA, specifically %MVPA and motivation for PA (Table 2, ρ = 0.187, *p* = 0.012), which is similar to recent work utilizing PA with EMA^7^. However, when accounting for intra-subject variability using mixed-effects models, this relationship is found to be not significant (Table 3a, *p* = 0.351). This is in contrast to our expectation that PA itself can enhance intrinsic motivation if it satisfies autonomy, competence, and relatedness needs^30^. This result implies that other factors influence motivation for PA and that interventions that only focus on increasing PA may not provide lasting intrinsic changes in motivation. Without changes in motivation, the likelihood of interventions to result in post-interventional behavioral change decreases.

Several studies have established relationships between PA and PF, suggesting a possible role in building and maintaining motivation for PA. Indeed, while correlation analysis did not find strong evidence to support this, the mixed-effects models (Table 3b and 2c) find that dynamic balance (TUG score) but not lower limb power (STS score) was a significant predictor of motivation for PA. This result aligns with previous studies establishing the relationship between PA and PF, where the functional ability of the older adult individual plays a role in engaging in PA.^29,32,51,52^ The lack of a significant relationship between motivation and STS may be due to the non-specific nature of the STS assessment, as the result is limited to a discrete number of repetitions. This result provides guidance for future interventions to focus on building PF to create stable increases in motivation to maintain recommended levels of PA. ICCs ranged from 0.38 to 0.49, indicating that a substantial amount of variability in motivation scores is due to repeated measures within individuals. Marginal R^2^ values ranged from 0.033 to 0.131, suggesting that fixed effects had modest explanatory power. In contrast, Conditional R^2^ values ranged from 0.403 to 0.554, indicating that accounting for individual and daily differences considerably improved model fit.

As discussed earlier, different types of motivation have varying effects on PA across age groups. Here, we consider PA as a driver of intrinsic motivation, supported by previous literature. However, evaluating PF considers its contribution to introjected and external motivation, as previous studies have evaluated PF in different motivational profiles^53^. We hypothesize that introjected motivation can be linked to avoiding the shame of decreased PF, while receiving the reward of improved dynamic balance can drive external motivation^54,55^. The results presented here instead focus on rating amotivation, referring to the absence of motivation. In this way, we do not directly assess more self-determined and autonomous types of motivation but focus on how PA and PF affect the presence of motivation. Our findings support the COM-B model for behavior change in physical activity, revealing capability as a driver of motivation of PF. By understanding the basic patterns of how PF can increase motivation, we can inform interventional models to frame interventions that improve feelings of satisfaction by increasing PF.

The limitations of our study include the sample composition and method of EMA delivery. The predominantly female sample reflects the population of older adults that attends community centers and suggests that EMA may preferentially be implemented in older women than men. However, low-income older women disproportionately experience a greater rate of falls, increased fear of falling (FoF), and balance impairment related to osteoporosis and menopause^13,56,57^, reflecting the significance and potential for these results. Additionally, outside of age and gender our mixed effects models did not consider the effects of confounding factors such as chronic diseases and medications, which contributes to much of the variance relating to random effects. Due to the wide variety of medical conditions and medications and their effect on PA and PF, we did not include them separately and instead encompassed them within the tested drivers. The method of EMA delivery using MetricWire had many advantages, including broad compatibility with phones used by low-income older adults. However, the method of collecting information about motivation for PA relied on a single question, answered on a sliding scale, as opposed to validated multi-item questionnaires. While this improved compliance by providing older adults with a quick, easy interface to enter and report data over the course of a week, future research should evaluate the validity of the single question by comparison to questionnaires such as the Exercise Motivations Inventory. The data collection period of one week is an additional limitation as it may not be sufficient to capture long-term variations in motivation. However, we are able to use the shorter period to evaluate relationships with PA and PF.

We present the predictive effects of the PF measure dynamic balance, on motivation for PA. By providing EMA in real-time over the course of a week, the results offer a view into the subjective perspectives of older adults, harnessed alongside accelerometers.

## Conclusions

Currently, many fall risk and exercise implementation interventions focus on reframing subjective perspectives and improving adherence to PA recommendations separately, rather than focusing on the factors that may influence both. The data from this study revealed the feasibility of older adults for mobile delivery of EMA for assessing subjective attitudes toward PA. Additionally, the results of this research include broader implications to assess both baseline PA and PF before tailoring interventions for older adults to maximize and monitor motivation for engagement in healthy behaviors such as the recommended levels of PA. This study provides a first step to understanding the predictive factors of motivation for PA, thus informing the inclusion of subjective information in future interventions.

## Figures and Tables

**Table 1.** Descriptive statistics for study variables.

Variables	Type of Variable	Mean (SD)
Motivation for PA	Dependent (response)	4.436 (0.266)
%MVPA	Independent (predictor)	0.033 (0.032)
TUG		12.79 (4.090)
STS		12.21 (5.465)

**Table 2.** Spearman’s correlations between motivation for PA, %MVPA, and PF.

Variables	Spearman’s ϱ	p-value
Motivation - %MVPA	0.187	0.012
Motivation – TUG	−0.157	0.036
Motivation – STS	0.200	0.007

%MVPA = Observed Moderate-to-Vigorous Physical Activity. TUG = Timed Up and Go scores, in seconds. STS = Sit to Stand score.

**Table 3.** The effect of PA and PF on motivation for PA. (2a) observed PA, as represented by % Moderate-to-Vigorous Physical Activity (%MVPA) did not significantly predict motivation for PA. However, of the PF measures, (2b) dynamic balance as represented by Timed-Up-and-Go (TUG) score, but not (2c) lower limb power as represented by the Sit-to-Stand (STS) score, significantly predicted motivation. Random effects of Day and Participant were specified to account for person-level and day-level variance. σ2—residual variance at level 1 (observation). ICC—intraclass correlation. Bolded p-values represent significance as defined as p-value < 0.05. Empty cells refer to terms that were not included in the model.

	Outcome: Motivation for PA					
	Model A		Model B		Model C	
*Predictors*	*β*	*p-value*	*β*	*p-value*	*β*	*p-value*

(Intercept)	4.51	**<0.001**	4.26	**<0.001**	3.91	**<0.001**
%MVPA	0.76	0.351	---	---	---	---
TUG Score	---	---	−0.03	**0.001**	---	---
STS Score	---	---	---	---	−0.01	**0.179**
Age	−0.00	0.898	0.01	0.297	0.00	0.459
Sex	−0.11	0.278	−0.15	0.181	−0.11	0.319

σ^2^	0.05		0.04		0.04	
τ_00 Participant_	0.03		0.04		0.04	
τ_00 Day_	0.00		0.00		0.00	
ICC	0.38		0.49		0.48	
Marginal / Conditional R^2^	0.033 / 0.403		0.131 / 0.554		0.054 / 0.504	
